# Diagnosis of an intestinal mucormycosis ‘fungus ball’ located with PET/CT with [^18^F] FDG-PET/CT

**DOI:** 10.1186/s41824-019-0068-0

**Published:** 2019-12-12

**Authors:** Franklin Gallo, Lavinia Vija, Sophie Le Grand, Nada Moukarbel, Koen Mortele, Erwan Gabiache, Frédéric Courbon, Suzanne Tavitian, Lawrence O. Dierickx

**Affiliations:** 1 Department of Radiology, Institut Claudius Regaud, Institut Universitaire de Cancer de Toulouse-Oncopole, 1, av Irène Joliot-Curie, 31059 Toulouse, France; 2 Department of Nuclear Medicine, Institut Claudius Regaud (ICR), Institut Universitaire du Cancer de Toulouse-Oncopole (IUCT-O), 1, av Irène Joliot-Curie, 31059 Toulouse, France; 3 Department of Haematology, Centre Hospitalo-universitaire (CHU) de Toulouse, Institut Universitaire du Cancer de Toulouse-Oncopole (IUCT-O), 1, av Irène Joliot-Curie, 31059 Toulouse, France; 4 Department of Pathology, Centre Hospitalo-universitaire (CHU) de Toulouse, Institut Universitaire du Cancer de Toulouse-Oncopole, 1, av Irène Joliot-Curie, 31059 Toulouse, France; 5 Department of Radiology, Beth Israel Deaconess Medical Center (BIDMC), 330 Brookline Ave, Boston, MA 02215 USA

**Keywords:** Intestinal mucormycosis, PET/CT, FDG, Fungal infection

## Abstract

Mucormycosis is a life-threatening infection with most commonly rhino-orbital-cerebral and pulmonary syndromes that mostly occurs in immunocompromised patients. FDG-PET/CT emerged as a sensitive non-invasive tool to identify systemic mucormycosis. We present a 59-year-old woman for whom a PET/CT with ^18^F-FDG was performed in search of a primary location of mucormycosis with non-contributive conventional workup. A large left abdominal mass was seen, compatible with a fungus ball, with intense parietal uptake and without any central uptake. The localization of the infection provided a target for surgery and permitted to adapt the therapeutic strategy. After resection, the final diagnosis was consistent with mucormycosis. To our knowledge, this is the first report of a PET/CT image with FDG showing an intestinal fungus ball. PET/CT with ^18^F-FDG may contribute to the management of patients with fungal infections of unknown origin.

## Background

Mucormycosis, previously called zygomycosis, is a life-threatening infection that mostly occurs in immunocompromised patients. It is a rare human infection reflecting the effectiveness of the immune system as all humans have ample exposure to the fungi during day-to-day activities and because infections occur almost always in the presence of an underlying compromising condition (diabetes, hematologic malignancy). The infection involves most commonly the facial sinuses, with possible extension into the brain, and the lungs presumably because of the inhalation of the spores into the paranasal sinuses. Other rarer deep localizations have been described (Barnes et al. 2017; Chinen et al. 2009; Marutsuka et al. 2013; Rashid et al. 2019; Ahmad et al. 2019; Forrester et al. 2015). The gastrointestinal tract is an unusual location, and infection probably occurs because of the ingestion of spores. The most common site seems to be the stomach followed by the colon (Agha et al. 1985). Treatment involves a combination of surgical debridement of the involved tissues and antifungal treatment. The diagnosis is difficult to establish as it depends on the identification of organisms in the tissue by histopathology with culture confirmation. However, culture often yields no growth. This explains why rates of delayed diagnosis and mortality are still high (Farmakiotis and Kontoyiannis 2016). Thus, in an appropriate clinical setting, the clinician often starts an empirical treatment for mucormycosis because early initiation of therapy is crucial. However, to maximize the outcome, surgery is essential which requires the localization of the primary infection. ^18^F-FDG can be used for its nonspecific uptake in infectious, inflammatory, and neoplastic processes. FDG-PET/CT emerged as a sensitive non-invasive tool to identify systemic mucormycosis (Dang et al. 2012; Liu et al. 2013; Altini et al. 2015; Song 2019), which can serve as a new diagnostic modality and have a significant impact on the therapeutic management.

## Case presentation

**Fig. 1:**
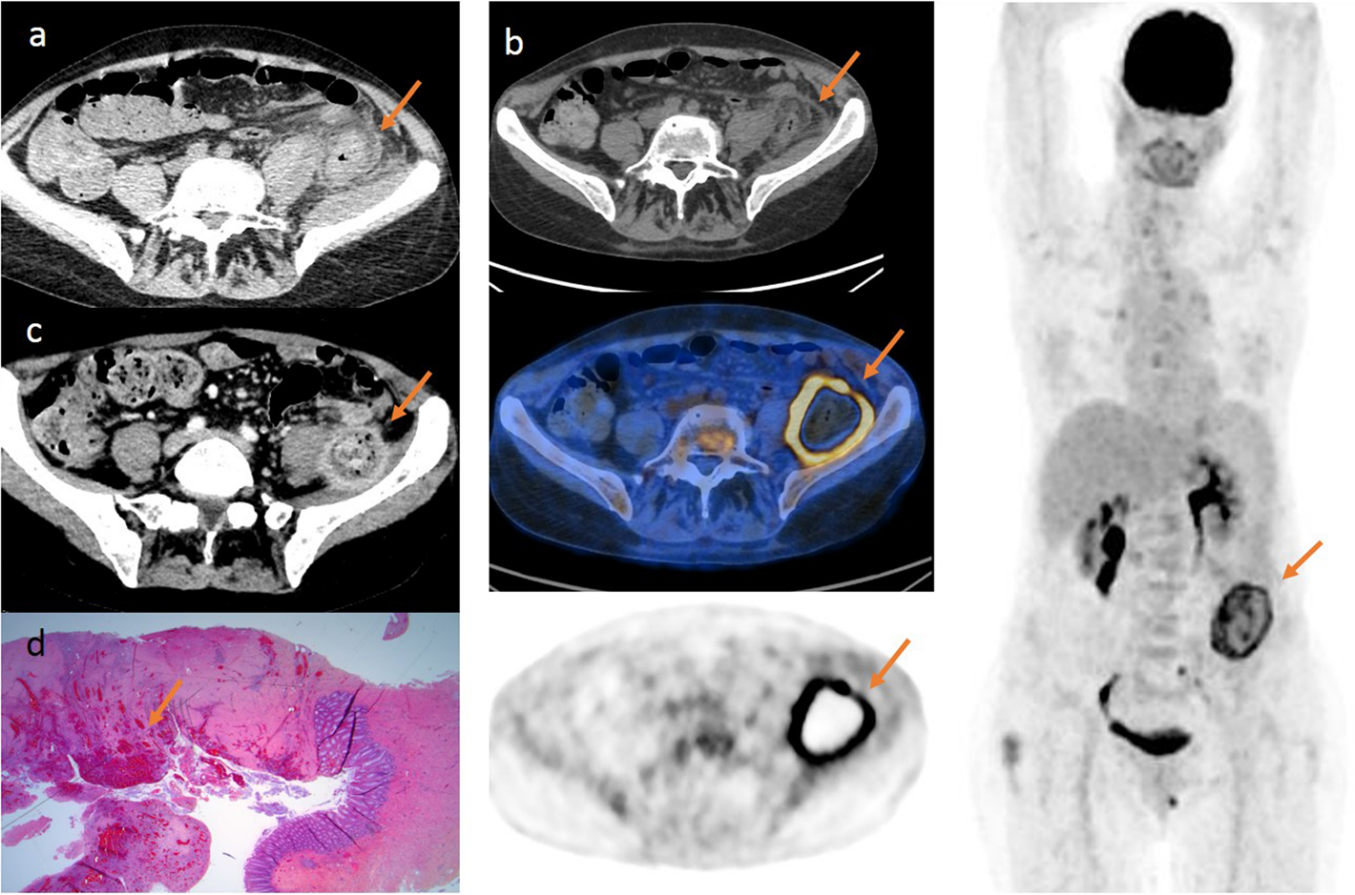
Intestinal mucormycosis “fungus ball” located with [^18^F]-FDG-PET/CT. **a** CT image on 3 November 2018 without contrast injection of aspecific colitis as shown by the orange arrow. **b** PET/CT image on 15 November 2018 of the intestinal fungus ball: left column: top panel: CT axial view without contrast; middle panel: fused PET/CT image; bottom panel: PET axial view; the right column shows a whole body volume–rendered image. The orange arrows show the intestinal fungus ball. **c** CT with the injection of contrast on 19 December 2018 showing a fibrotic inflammatory mass with necrotic center adhering to the iliopsoas muscle and encompassing the descending colon as shown with an orange arrow. **d** 03 January 2019: histological exam of intestinal resection. Coloration used: hematoxylin-eosin; magnitude: X1.25

We present herein a case of a 59-year-old woman initially diagnosed with acute myeloid leukemia. Post induction chemotherapy, this pancytopenic patient developed a persisting fever despite broad-spectrum antibiotics. A thoracoabdominal CT (a, arrow) was performed, without IV contrast because of renal failure, and showed features of aspecific colitis with wall thickening, thickening of the fascial planes, and infiltration of the pericolic fat and the iliopsoas muscle (Fig. 1). PCR blood analysis for mucor demonstrated positivity, and an isavuconazole treatment was started. Subsequent thorax and sinus CT was negative. Ten days after the first CT (a), a PET/CT with ^18^F-FDG (b, arrow) was performed in search of a primary location for eventual resection. Images were acquired on a GE Discovery IQ PET/CT (GE Healthcare, Milwaukee) 65 min after injection of 138 MBq of FDG. A large left abdominal mass was seen, compatible with a fungus ball, with intense parietal uptake (SUV max bw 8.81) and without any central uptake and in which the colon descendens seems to enter and exit without deformation. A month later, a second pre-operative CT with IV contrast (c, arrow) was performed and showed a fibrotic inflammatory mass with necrotic center adhering to the iliopsoas muscle and encompassing the descending colon. The final histological diagnosis after resection was a chronic ischemic ulcerated enterocolitis with fistulation and associated abscess, consistent with mucormycosis as it typically invades blood vessels with ischemia and loss of blood supply (d, arrow) (Fig. 1). No hyphae were seen, and culture for mucormycosis remained negative. The patient recovered well from the surgery. On a hematological plan, the patient is actually considered in complete remission. An allogenic stem cell transplantation was performed after the digestive surgery.

## Discussion

Along with clinical, laboratory, and histopathological data, imaging plays an important role in the diagnosis and management of mucormycosis. Even though the most common primary locations are usually well located with a standard thorax and sinus CT, other deep locations may present a problem. This is particularly important in this setting as resection of the fungus ball is necessary to complete the treatment. ^18^F-FDG-PET/CT has proven to be valuable in providing complementary information concerning the primary location and the extent of the fungal disease.

## Conclusion

To our knowledge, this is the first report of a PET/CT image with FDG of an intestinal fungus ball which permitted to localize the disease, which provided a target for surgery, and to adapt the therapeutic strategy. PET/CT with ^18^F-FDG may contribute in the management of patients with fungal infections of unknown origin.

## Data Availability

The images and biopsy samples remain available in our database.

## Abbreviations

^18^F-FDG-PET/CT: ^18^fluorine fluoro deoxyglucose positron emission tomography/computed tomography
